# Sexual Health and Drug Use Prevention for Black Girls (The Dreamer Girls Project): Protocol for an Intervention Development

**DOI:** 10.2196/45007

**Published:** 2023-08-09

**Authors:** Ijeoma Opara, Cora Gabriel, Beatriz Duran-Becerra, Keosha Bond, Ashley V Hill, Sydney Hussett-Richardson, Courtnae Alves, Trace Kershaw

**Affiliations:** 1 Yale School of Public Health New Haven, CT United States; 2 City University of New York School of Medicine New York, NY United States; 3 University of Pittsburgh Pittsburgh, PA United States; 4 Columbia University School of Public Health New York, NY United States

**Keywords:** HIV prevention, drug use prevention, Black girls, youth, formative, Black, girl, adolescent, drug abuse, substance abuse, HIV, prevention, patient education, education intervention, sexual transmission, sexually transmitted, sexually transmitted disease, STD, sexually transmitted infection, STI, sexual health, health promotion, ethnic, minority

## Abstract

**Background:**

Substance use among adolescent girls is associated with numerous risk characteristics, including engaging in sexual risk behaviors, which can lead to HIV and sexually transmitted infection (STI) diagnoses. This is an important phenomenon to target as there is a significant race-gendered paradox that occurs when Black girls use and misuse drugs. When misuse occurs among this group, they are more likely to face harsher consequences and worse health outcomes than boys and other ethnic-minority girls. Therefore, there is a need to understand the risk and protective factors of drug use and sexual risk behaviors among Black girls and develop a robust intervention that can cater for this group.

**Objective:**

We propose the development of a strengths-based prevention education intervention for Black girls between the ages of 13 and 18 years to promote protective factors.

**Methods:**

A sequential, mixed methods study will be conducted, and we will use the first 3 steps of the ADAPT-ITT (assessment, decision, adaptation, production, topical experts, integration, training, testing) framework to begin the development of the intervention. Three aims will be described in this protocol. First, aim 1 is to explore sociocultural risk and protective factors among Black girls between the ages 13 and 18 years in drug use and HIV/STI prevention using focus group methodology and surveys. We will conduct at least 10 focus groups to include up to 75 Black girls or until we reach saturation. Our target sample size for the quantitative portion of the study will be 200 participants. Aim 2 will focus on deciding upon an intervention based on findings from aim 1 and forming a youth advisory board to guide intervention development. Aim 3 will be to conduct a pretest of the intervention with the youth advisory board to determine if the intervention is feasible and will be accepted by Black girls.

**Results:**

The study is part of a 2-year research pilot study award from the National Institutes of Mental Health. Data collection for this study began in October 2021. For aim 1, data collection is 95% complete. We expect to complete all data collection for aim 1 on or before May 30, 2023. Study activities for aim 2 are occurring simultaneously as data are being collected and analyzed and will be completed in the summer of 2023. Study activities for aim 3 will begin in the fall of 2023.

**Conclusions:**

This study will be one of the few interventions that address both sexual health and drug use together and cater to Black girls. We anticipate that the intervention will be beneficial for Black girls across the nation to work on building culturally appropriate prevention education and building peer social supports, resulting in reduction or delayed substance use and improved sexual health.

**Trial Registration:**

ClinicalTrials.gov NCT05014074; https://clinicaltrials.gov/ct2/show/NCT05014074

**International Registered Report Identifier (IRRID):**

DERR1-10.2196/45007

## Introduction

Despite substantive literature on the relationship between adolescent drug use and sexual risk behavior, gaps in prevention research may continue to place Black girls between the ages of 13 and 18 years in the United States at risk. Historically, girls of all racial and ethnic groups in the United States have had lower drug use rates than boys [1]. However, over the past decade, the prevalence of smoking cigarettes, drinking alcohol, and using illicit drugs such as marijuana has increased among adolescent girls, and specifically increased as they get older [2,3]. In the past decade, Black girls have begun to engage in substance use more frequently and at rates similar to their male counterparts [2]. This is concerning because drug use among adolescent girls is linked to a variety of risk factors, including engaging in sexual risk behaviors [4,5]. According to the 2019 Centers for Disease Control and Prevention Youth Risk Behavior Survey, while cigarette use is almost nonexistent for this group, 38.6% of Black girls admitted to using electronic vapor products, with 17% of them using within the past 30 days. Similarly, 37% of Black girls in the study used marijuana, with 24.8% using marijuana within the past 30 days [2]. The rates of marijuana among Black girls have also increased significantly from early to late adolescence [6,7]. Black girls may face a significant “gender paradox” where although they are less likely to use substances than boys and other ethnic-minority girls, they tend to face more negative health consequences from use [8].

Black girls are more at risk of being persuaded to engage in sexual risk behavior such as drinking alcohol or using drugs before having sex, having sex without a condom, and having multiple sexual partners than their White counterparts [9]. These behaviors may be the direct consequence of belonging to multiple marginalized groups (eg, race, ethnicity, class, and gender), resulting in intersecting multiple levels of oppression [10]. Such behaviors may arise from the historical and contextual factors of trauma, gendered racism, and sexual exploitation that are pervasive in the lives of Black girls [11-13]. Lack of discussion and knowledge of HIV/AIDS prevention strategies, limited or nonexistent community-based preventive resources, and gender- and racial-specific behavioral interventions in urban, underresourced communities can significantly contribute to increases in sexual risk behaviors [14,15]. Sexual health disparities and drug misuse that affect Black girls not only produce poor health outcomes but can also contribute to educational inequities [16], increases in school suspension rates [17], criminal involvement [18], income inequality [19], and sexual victimization [20].

The multiple marginalized identities of Black girls place them at increased risk of early sexual debut (ie, having sex at or before the age of 14 years), making them more susceptible to condomless sex and having partners that encourage sexual risk behaviors and making them more likely to be sexually abused, which increase their risk for HIV and sexually transmitted infections (STIs) [14,21]. In one study, the authors found that among sexually active Black girls between the ages of 14 and 19 years, 48% were diagnosed with at least 1 sexually transmitted infection [22]. Being diagnosed with an STI places individuals at an increased risk of contracting HIV [23]. In addition, adults living with HIV/AIDS are more likely to engage in risky sexual behavior during adolescence [24]. As Black women currently represent 61% of women living with HIV/AIDS [25], it is crucial to intervene with Black girls in order to provide them with prevention strategies that can be used as they transition into adulthood.

Although HIV and drug abuse prevention initiatives for adolescents exist, many are homogeneous, aim to be generalizable in nature, and do not consider unique racial and gender differences in risk and protective factors [26]. Furthermore, there are very few integrated HIV and drug abuse prevention interventions for Black girls [27]. As age can limit mobility geographically, which may then contribute to the further marginalization of Black girls due to limited power and dependency on others [28]. Multiple contextual factors that are present in the lives of Black girls are often ignored, thus leaving many strategies that are embedded in prevention interventions, such as educational components that do not center the lived experiences of Black girls, not helpful for this group [27]. Researchers have called for HIV and drug use prevention research that uses an intersectional framework to examine how multiple identities interact to have an impact on attitudes and behaviors [29,30].

This study protocol is describing the formation of a mixed methods study called “The Dreamer Girls Project,” which will first understand the challenges and protective factors present in the lives of Black girls related to substance use and sexual health and then use the findings to develop a strengths-based prevention intervention. The study team will work collaboratively with a youth advisory board and use formative data collection to inform the development of the intervention. The study aims are shown in Textbox 1.

This study has the potential to provide new information about risk and protective factors for Black girls and strengths-based strategies that can be infused in HIV, STI, and drug use prevention for Black adolescents between the ages of 13 and 18 years.

**Study aims.**

- Aim 1: Understand sociocultural risk and protective factors among Black girls between the ages 13 and 18 years in drug use and HIV/STI prevention using focus group methodology and surveys.
  Aim 1a: Quantify the prevalence rates of drug use and behaviors associated with HIV risk among Black girls aged 13-18 years.
- Aim 2: Decide upon an intervention based on findings from aim 1 and form a youth advisory board to guide intervention development.
- Aim 3: Assess the feasibility of the intervention through theater testing.

## Methods

### Conceptual Framework

The study will use intersectionality theory as a theoretical framework to guide the formative phase and overall development of the intervention [31-33]. Our usage of intersectionality theory will incorporate a culturally relevant perspective that includes the voices of Black girls, allowing them to redefine themselves, include discussions around protective factors that they themselves perceive as beneficial in their lives, and celebrate supportive structures that nurture their ability to be resilient. An intersectional perspective, therefore, provides a unified lens through which to view health disparities and develop interventions that are more responsive to race, ethnicity, gender, age, and class [28]. Intersectionality functions as a framework in this study to understand the experiences of Black girls, who possess a different social location than Black women based on their age and cognitive and psychological development. In addition, because an intersectional perspective can be strengths based, it moves the blame away from individual behaviors to systemic and contextual factors that have historically placed Black girls at risk. The framework considers the cultural and historical context of Black girls that influences their susceptibility and exposure to drug use and sexual risk behaviors, as well as highlights the significance of systemic racism, classism, and misogyny within the United States as contributing factors to their exposure. Such a framework has been useful not only in designing and guiding social programs but also in developing ethnic, racial, gender, and class-responsive research for girls. It is to be acknowledged that Black girls are not a homogenous group, which means that significant within-group differences will exist. Therefore, the use of intersectionality theory as a framework allows for our study team to account for those differences in order to make interventions more appropriate to the experiences of Black girls [31].

### Study Design

We will conduct an exploratory, sequential mixed methods study. The protocol is registered on ClinicalTrials.gov (NCT05014074) and was approved by Yale University Institutional Review Board.

### Eligibility

Adolescent girls who identify as (1) Black, (2) identify as female, (3) are between the ages of 13 and 18 years, (4) can speak and understand English, and (5) live in the United States will be allowed to participate in this study.

### Ethics Approval

All procedures performed in studies involving human participants are in accordance with the ethical standards of the institutional and national research committee, the 1964 Helsinki Declaration and its later amendments, or comparable ethical standards. The study was funded by the National Institute of Mental Health (R25MH087217) and approved by the Yale University Institutional Review Board in 2020 (IRB-2000026660). Due to the sensitivity of the research questions, we requested that written parental consent be waived for the young participants. All youth who participate in the study (including focus groups and completing surveys) will receive a youth information sheet and parents and guardians will receive a parent information sheet. Youth must give verbal consent in order to participate in the study. We will obtain informed consent from all girls who meet the eligibility criteria and want to participate before they are enrolled in study activities.

### Recruitment

Purposive sampling for focus group recruitment will be used to ensure a representation of different subgroups of Black girls. The research team has relationships with community-based organizations in New Jersey and organizations that serve Black girls across the nation. Flyers will be given to program administrators at these organizations to disseminate to interested participants. Flyers for the recruitment of focus groups will also be posted on social media sites such as Facebook, Instagram, and Twitter. All girls who are interested in being a part of the focus groups will have to complete an eligibility form on Qualtrics (Qualtrics International Inc), which will ask for demographic information and contact information. Only girls who identify as Black and female, are between the ages of 13 and 18 years, and live in the United States will be allowed to participate. Research staff will contact eligible participants and will inform them of the purpose of the study and will screen them to confirm that they are eligible to participate based on inclusion criteria. Potential participants will be told that all data will be kept anonymous and confidential and that a monetary incentive of US $30 will be provided for participants who complete the focus group. Potential participants for the qualitative component of this study will also be informed that their responses to questions will be recorded. Upon confirmation of their desire to participate, they will be given a date, time, and Zoom (Zoom Corp) link if web-based or a physical location if appearing in person. The institutional review board has approved this study and agreed to waive parental consent due to the sensitivity of the topic. Youth assent will be obtained verbally. All youth and parents and guardians will be provided an information sheet regarding their daughter’s participation in the study. All participants and parents and guardians of participants younger than 18 years will be told that they can opt out at any time during the study. Community organizations who are able to provide us with space for in-person focus groups will receive honorariums. Recruitment for survey data collection will involve sharing the survey link on various social media platforms and community organizations that serve Black girls. Only girls who identify as Black and female, are between the ages of 13 and 18 years, and live in the United States will be allowed to complete the survey. All participants who completed the survey were compensated with a US $10 gift card.

### Procedures

A semistructured interview guide using intersectionality theory will be developed to explore drivers of substance use, social supports necessary to promote the prevention of substance use and supports necessary to reduce HIV risk behaviors. The study principal investigator (PI) and first author of the protocol (IO) is an expert in using intersectionality theory as a framework for studies involving Black girls. More specifically, the study PI has published studies that used intersectionality theory as a framework in analyzing quantitative and qualitative data [10,34,35] and teaching courses using an intersectionality framework [36]. After the interview guide was developed, another expert who serves as a coauthor of this protocol (KB) will review and revise the guide to ensure that questions use an intersectionality framework. KB is an expert on intersectionality theory and qualitative methodology with Black cisgender and transgender girls and women. Collectively, the study team including the study PI, who has several years of experience in designing studies for Black girls, will develop questions to ensure that girls across the development age span of 13 to 18 years would be able to understand the questions.

Data collection will involve in-depth focus groups to be conducted in a group setting at a community partner’s location sites (mainly in New Jersey due to the study team’s relationships with New Jersey–based community organizations) and web-based groups will be conducted on Zoom. Research in web-based settings has been increasing due to the COVID-19 pandemic and may continue to be a major platform to connect with participants. Research conducted in web-based settings has been established as effective venue for research on multiple content areas [37]. Efforts will be made to place participants into groups that are separated by demographic variables such as age or religion (ie, aged 13-15 and 16-18 years) as it is recommended to group individuals with similar life experiences for optimal focus group dynamics. Each focus group will be audio recorded, transcribed by trained research assistants, and entered into NVivo 11 (QSR International), a qualitative data analysis software package. Focus groups will aim to uncover how Black girls view substance use and sexual risk behavior in their community. Questions that will be discussed in the focus groups are (1) How do race, gender, and class impact views on drug use and sexual risk behavior among Black girls? (2) How do Black girls view their identity and expectations that they perceive from society? (3) What are the risk factors of engaging in drug use and sexual risk behavior that are specific to Black girls? (4) What are protective factors and what do they look like? and (5) What would a successful prevention intervention look like for Black girls in various communities? While 3 or 4 focus groups are recommended for saturation [38], we aim to conduct at least 10 focus groups or until no new data emerge from focus group discussions.

### Training of Interviewers

Interviewers will be research staff who identify as Black women, trained by a qualitative methodology expert (KB) on how to engage and conduct focus group interviews with Black girls. The training will involve discussions around being aware of power dynamics that may emerge during the interview in order to facilitate the focus groups effectively. In addition, interviewers will be challenged on assumptions and biases that they may have of Black girls in order to assure that data collection is conducted in an objective and respectful manner that honors Black girls. All facilitators and cofacilitators must identify as Black women, as there is evidence of the importance of racial and gender matching when conducting research with vulnerable and marginalized groups such as Black girls [39].

### Data Analysis

#### Aim 1: Understand What Environmental and Sociocultural Factors Influence Drug Use and Sexual Risk Behavior Among Black Girls

##### Aim 1a. Determine Prevalence Rates of Substance Use and Sexual Risk Behaviors Among a Sample of Black Girls

Within this aim, we will use a sequential exploratory study design [40] to understand risk and protective factors that are specifically mentioned by Black girls through engaging in mixed methodologies. Sequential exploratory design is used when a study team wants to follow-up or explains qualitative findings with quantitative analysis. This 2-phase approach is particularly useful for our study as we are interested in understanding the unique perspective of Black girls and want to develop an intervention catered for this population.

First, we will use focus group methodology, which will allow the study team to receive insight from Black girls themselves about what they perceive as the challenges but also strengths that may be present in their environments, families, and communities involving drug use and sexual health. Focus group methodology is useful in this phase as it not only provides a rich source of data but also allows the researcher to examine the interaction among participants [41]. We will aim to conduct at least 10 focus groups, with a goal to obtain a sample size of 75 Black girls or until data saturation is reached. We acknowledge that this is a large sample size. However, since we will be interviewing Black girls from across the nation, we believe that having a large sample size will give us richness in the data and allow us to explore differences and similarities across the groups.

After focus groups are conducted and before the analysis begins, facilitators of the focus groups will participate in debriefing meetings. These meetings will provide a space for facilitators to process each focus group and discuss possible issues of countertransference that may have occurred. This is necessary as we want to minimize any potential to influence the results of the study. In addition, the debriefing meetings provide facilitators the space to discuss their feelings and emotions that arose during the groups. The data analysis team will include at least 3 research assistants and the study PI who has experience in analyzing qualitative analysis and leading qualitative studies. To establish confirmability the research team will code all of the transcripts. Data will be coded using NVivo version 11. Data from the interviews will be first analyzed by interviewers using open coding, whereby concepts were identified and labeled as they emerged from the data and across the focus groups. Each member of the research team will read and reread all the transcripts and develop initial codes in isolation. During weekly research meetings, the initial codes will be reviewed and discussed. Using multiple coders provides insight into finding consistency and minimizing researcher bias [42]. The team will triangulate codes, notes, and memoranda of analyses until >95% substantive agreement is achieved. Prevalent codes (based on the frequency in which a code is used by participants across and within groups) will be grouped by similarity and relevance into themes and subthemes. To increase the rigor of the study, the researchers intend to use prolonged engagement, regular team debriefings, and member checking with participants [43]. Participants of the focus groups will be asked if they are interested in being a part of the youth advisory board. The youth advisory board will be developed for the sole purpose of confirming the findings and developing the intervention. We will aim to recruit 8-10 youth upon completion of the qualitative portion of the study.

##### Aim 1b: Quantitative Data Collection

Based on the findings from the qualitative focus groups and the use of a sequential exploratory study design, data will be analyzed rapidly [44] and inform the development of the survey that will be administered to Black girls. In order to accurately perform rigorous statistical analysis tests such as structural equation modeling, we proposed a sample size of 200 girls as performing structural equation modeling on data requires a sample size of at least 150 [45]. We anticipate including standardized measures such as 30-day drug use and sexual risk behavior measures, adapted by the Centers for Disease Control and Prevention Youth Risk Behavior Survey [2]. In addition, we will include measures pertaining to ethnic identity, sexual orientation racial discrimination, and gender identity.

#### Aim 2: Decide Upon an Intervention Based on the Findings From Aim 1 and Form a Youth Advisory Board to Guide Intervention Development

Because the goal of this study is to obtain information from Black girls in order to develop an intervention for them, we will use an implementation science framework that centers the voices of participants. We will use ADAPT-ITT (assessment, decision, adaptation, production, topical experts, integration, training, testing) [46], an implementation framework that has been commonly used to adapt HIV interventions. The ADAPT-ITT model involves eight steps: (1) assessment of priorities, (2) decisions on adapting, (3) administration of intervention, (4) production of adapted version, (5) topical experts, (6) integration of feedback from topical experts, (7) training staff to implement, and (8) testing the adapted intervention. Because of the nature and funding restrictions of this pilot study, we will only complete the first 3 steps.

For aim 1, the assessment step requires researchers to obtain a comprehensive understanding of the target population. Next, for aim 2, a youth advisory board of 8-10 Black girls will be formed and will select the interventions that they will decide to adopt or adapt. The decision phase will be guided by themes that arose from the focus group and quantitative findings, in order to assure we will be choosing interventions that best meet the needs of Black girls. Our youth advisory board will first be presented with common HIV prevention interventions catered toward Black girls and women such as SiHLE (Sistering, Informing, Healing, Living, and Empowering) [47] and will be asked to vote on each of their strengths and weaknesses. We will then work with our study team, guided by the youth advisory board to either modify components of a particular intervention or create our own intervention. The study team has conducted a systematic review of the most commonly used HIV and drug use interventions that include large samples of Black girls and has expertise in the various components of interventions for this group [27].

#### Aim 3: Assess the Feasibility of the Intervention Through Theater Testing

Using the ADAPT-ITT framework, we will enter into step 3, which is the adaptation phase. This phase involves using an innovative pretesting methodology known as theater testing to adapt the evidence-based intervention that the study team and youth advisory board decide upon. Theater testing is a type of pretesting methodology that is commonly used to test products, such as television advertisements, videos, print advertisements, and public service announcements [48]. Using this methodology, participants, which will be the youth advisory board, will be invited to laboratory or community site to respond to a demonstration of a product (ie, of the adapted intervention). At the end of the demonstration, participants will receive a questionnaire and answer questions designed to gauge their reaction to the product. An important strength of this methodology is the opportunity to obtain reactions to messages, concepts, and visual materials in a relatively short period. Furthermore, this methodology closely resembles what is experienced by the target population; thus, an accurate assessment of their reactions to the product can be obtained.

## Results

The study is part of a 2-year research pilot study award that received funding from the National Institutes of Mental Health. Data collection for this study began in October 2021. The remaining year will focus on completing data collection, analysis, and dissemination and developing the intervention components. We have published 1 manuscript based on the formative work for the development of this intervention, which was a systematic review of sexual health and drug use prevention interventions for Black girls [27]. Moreover, data collection for aim 1 is 95% complete. We expect to complete all data collection for aim 1 on or before May 30, 2023. Recruitment for aim 2 will begin in the summer of 2023.

## Discussion

### Anticipated Findings

The goal of this study is to understand the lived experiences of Black girls and obtain information that can be used to develop and pilot a race-specific intervention for this group. We anticipate that having Black girls co-design this intervention with the research team will have great potential to facilitate healthy behaviors and challenge negative stereotypes that impact Black girls’ decision-making over the course of the study and thereafter. This study has the potential to transform the way researchers work with Black girls and cocreate in order to develop sustainable solutions toward ending the HIV epidemic and addiction. The development of racial- and gender-specific intervention specifically for Black girls is essential, and developing interventions specific to a certain demographic has proven to produce successful results in health-promoting behaviors. For instance, one HIV prevention intervention tailored to the experiences and needs of Black women in Atlanta, Georgia was assessed for efficacy at improving condom use after 1 session. The authors found that at the 6-month follow-up women who participated in the session reported more frequent condom use and recent HIV screening [49]. Gender- and racial-specific interventions often move beyond individual risk behaviors and focus on social, environmental, and political influences on the well-being of women and girls. Specific focus on racial and gender pride, healthy relationships and healthy sexuality, reproductive justice, sexual communication, and empowerment around sexual refusal are just a few key aspects of effective interventions for Black women that may be tailored to Black girls [50]. The findings of this study will assist with identifying the elements of HIV prevention interventions specific to Black girls that can be applied broadly in the area to improve sexual health and HIV and STI prevention broadly. Given the current climate, interventions that address key factors such as racism, ethnic identity, and gendered racism must be integrated with intervention efforts for adolescent girls.

### Limitations

This is a formative sequential exploratory study design using mixed methods (qualitative data, cross-sectional survey data). A limitation of this study is that data will be collected via self-report and subject to response bias.

### Conclusions

The Dreamer Girls Project lessons learned will aim to provide real-world contributions to implementation science regarding the uptake of a combination of evidence-based prevention interventions guided by Black girls. The evidence from this study will be used for the preparation of a feasibility trial and a more robust and larger HIV, STI, and drug use prevention clinical trial specifically for Black girls.

## Abbreviations

ADAPT-ITT: assessment, decision, adaptation, production, topical experts, integration, training, testing
PI: principal investigator
SiHLE: Sistering, Informing, Healing, Living, and Empowering
STI: sexually transmitted infection

## Data Availability

The data sets generated during and analyzed during this study are not publicly available due to confidentiality reasons but are available from the corresponding author on reasonable request.
